# The Low-Renin Hypertension Phenotype: Genetics and the Role of the Mineralocorticoid Receptor

**DOI:** 10.3390/ijms19020546

**Published:** 2018-02-11

**Authors:** Rene Baudrand, Anand Vaidya

**Affiliations:** 1 Department of Endocrinology, Endocrine Hypertension and Adrenal Disease Program, School of Medicine, Pontificia Universidad Catolica De Chile, Santiago 8330074, Chile; baudrandrene@gmail.com; 2 Department of Medicine, Division of Endocrinology Diabetes and Hypertension, Center for Adrenal Disorders, Brigham and Women’s Hospital, Harvard Medical School, Boston, MA 02115, USA

**Keywords:** renin, low-renin, hypertension, mineralocorticoid receptor, genetics, aldosterone

## Abstract

A substantial proportion of patients with hypertension have a low or suppressed renin. This phenotype of low-renin hypertension (LRH) may be the manifestation of inherited genetic syndromes, acquired somatic mutations, or environmental exposures. Activation of the mineralocorticoid receptor is a common final mechanism for the development of LRH. Classically, the individual causes of LRH have been considered to be rare diseases; however, recent advances suggest that there are milder and “non-classical” variants of many LRH-inducing conditions. In this regard, our understanding of the underlying genetics and mechanisms accounting for LRH, and therefore, potentially the pathogenesis of a large subset of essential hypertension, is evolving. This review will discuss the potential causes of LRH, with a focus on implicated genetic mechanisms, the expanding recognition of non-classical variants of conditions that induce LRH, and the role of the mineralocorticoid receptor in determining this phenotype.

## 1. Introduction

The renin–angiotensin–aldosterone system plays a crucial role in volume, sodium, and potassium homeostasis. When renal hypofiltration is sensed, this hormonal system is activated via the secretion of renin, which catalyzes the generation of angiotensin I that is subsequently modified to generate angiotensin II. The potent vasoconstrictive properties of angiotensin II, in addition to its ability to stimulate the release of vasopressin and adrenal aldosterone secretion, help maintain arterial blood pressure and restoration of intravascular volume. Thus, physiologic activation of the renin–angiotensin–aldosterone system is characterized as a renin-dependent aldosteronism and serves to maintain blood pressure and volume in terrestrial mammals that evolved in a milieu of scarce dietary sodium intake. Despite this highly evolved physiology, low-renin hypertension (LRH) is currently a prevalent biochemical phenotype described in up to 30% of hypertensives, depending on age and race [1,2]. LRH is characterized by the physiologic suppression of renin, often in the context of intravascular volume expansion; however, there are many potential pathophysiological events that can result in hypertension with a low-renin phenotype that will be discussed in this review.

LRH has been described and investigated for nearly 50 years [3,4]. Shortly after Jerome Conn first described primary aldosteronism as a condition of aldosterone excess independent of renin [5], a phenotype of low renin activity in hypertension without overt hyperaldosteronism was described [3,4]. This phenotype was considered unique from primary aldosteronism and termed LRH, and subsequent studies described it as a condition more prevalent in individuals of African descent and elderly populations, who are also prone to salt-sensitive hypertension [6]. In subsequent decades, it was speculated that LRH might represent a heterogeneous mixture of etiologies that could include states of excessive mineralocorticoid receptor (MR) activation, as initially supported by LRH subjects manifesting low salivary sodium-to-potassium ratios and marked decreases in blood pressure in response to spironolactone and aminoglutethimide (an adrenocortical steroid synthesis inhibitor) [7,8,9].

The potential role of MR activation in LRH is important, since LRH and primary aldosteronism are similarly associated with higher risk for cardiometabolic events and death [10,11], and the availability of oral MR antagonists permits a potential targeted therapy. Further, the LRH phenotype displays familial aggregation, where several polymorphisms and novel genes have been described [12]. In one study, family membership explained 35% of variance in renin levels, far beyond the classic low frequency monogenic causes [12]. The composite of evidence suggests that the LRH is likely a heterogeneous admixture of disease states that all converge on a phenotype of suppressed renin activity and high blood pressure. These disease states include primary aldosteronism (PA), as well as conditions that manifest with low aldosterone levels, such as endogenous hypercortisolism, the syndrome of apparent mineralocorticoid excess (AME), atypical forms of congenital adrenal hyperplasia, and alterations in the activity of the mineralocorticoid receptor or epithelial sodium channel (Liddle syndrome). Consistent with this differential diagnosis, LRH has been shown to exhibit a bimodal distribution of aldosterone levels in population-based studies, supporting the existence of two broad categories of LRH: those with suppressed aldosterone and those with normal or elevated aldosterone [13]. New evidence suggests that beyond these general etiologic categorizations, diseases such as PA, hypercortisolism, AME, and perhaps even Liddle syndrome, may exist across a phenotypic continuum that extends from their overt and “classical” presentations to milder and “non-classical” phenotypes; the recognition of these expanding phenotypic continuums may improve our understanding of the pathogenesis and treatment of LRH and essential hypertension. Finally, environmental and nutritional factors, such as obesity, diabetes, and especially high dietary sodium intake, also play an important role in the development of the LRH phenotype. Putative mechanisms for these links include, new MR interactions, such as ligand independent activation of MR by sodium-RAC1 [14] and epigenetic modifications of LRH-related genes by DNA methylation (e.g., *HSD11B2* or adducin gene), histone modifications (e.g., epithelial sodium channel gene) or noncoding RNA (e.g., renin-angiotensin-aldosterone pathway genes) [15].

Herein, we will review known conditions that manifest with a phenotype of LRH, while focusing especially on postulated genetic mechanisms and the role of excessive MR activation.

## 2. Primary Aldosteronism

The most prevalent cause of LRH is primary aldosteronism (PA) [10,16]. PA is characterized by hyperaldosteronism that is independent of renin and angiotensin II (thus renin-independent aldosteronism) that results in excessive MR activation, increases intra-vascular volume and blood pressure, and results in renal, vascular, and cardiac disease, and higher mortality [10,11,16,17,18,19].

PA is considered the most common form of endocrine hypertension, with an estimated prevalence of 5–10% in the general hypertensive population, at least 6% in the primary care population, and up to 20% in the setting of resistant hypertension [11,17,20]. Since Conn’s initial description of the classical PA disease phenotype over 50 years ago, the understanding of the severity spectrum of PA and underlying genetics has greatly expanded [5,21]. First, human studies have shown that there is a broad spectrum of autonomous and renin-independent aldosteronism and MR activation; PA is not only a disease reserved for those with severe and resistant hypertension, rather can be detected in mild to moderate hypertension and also in normotension [20,22,23,24]. Normotensive individuals with higher aldosterone levels have a higher risk for developing hypertension, an association that is driven by normotensives exhibiting a PA phenotype: renin suppression with increasingly inappropriate aldosterone secretion [22,23,24,25]. Thus, it is becoming clearer that dysregulated autonomous aldosterone secretion that is independent of renin, even when it does not meet the classical definitions of overt PA, exists across a large continuum, and therefore, our strict categorization of PA may handicap clinical care by placing focus on only the most severe cases at the expense of ignoring milder disease [23,24,26,27]. Although clinical practice recommendations focus on defining PA using categorical thresholds [16,18], expert opinion is increasingly warning that “the strict definition of primary aldosteronism is no longer tenable,” and calling to “recognize the true prevalence of primary aldosteronism to include dysregulated aldosterone secretion and inappropriate aldosterone production” [28]. Second, excessive MR activation in PA contributes to significant cardiovascular and metabolic diseases, independent of blood pressure, such as diabetes and metabolic syndrome, stroke, myocardial infarction, left ventricular hypertrophy, atrial fibrillation, heart failure, and death [11,16,19,29,30,31]. Collectively, these two important observations have made it clear that recognizing and treating PA as early as possible is critical to prevent long-term adverse outcomes. Finally, our understanding of the pathogenesis of PA has dramatically improved with new genetic and histopathologic discoveries that have shed light on the mechanisms that might underlie PA. These advances will be discussed in more detail below.

The Endocrine Society clinical practice guidelines recommend identifying overt cases of PA by measuring the aldosterone-to-renin ratio (ARR) based on the clinical detection of severe or resistant hypertension, hypokalemia, an adrenal nodule, sleep apnea, or a family history of PA or early cardiovascular disease [16]. The most widely used cut-off for an aldosterone concentration is at least 15 ng/dL (and less frequently 10 ng/dL) with an ARR of at least 30 ng/dL per ng/mL/h [16]. This clinical approach lowers the risk for false positive screening results and, in general, is designed to detect overt and severe cases of PA. Alternatively, to recognize milder forms of PA, and to maximize early case detection in order to mitigate future cardiometabolic disease, more permissive screening criteria have also been proposed: a suppressed renin activity in the context of non-suppressed aldosterone (>6–9 ng/dL) consistent with an ARR >20 [16]. This latter approach may detect milder cases of PA, but will increase the risk of false-positive screening results, and consequently, potentially more costly and/or invasive medical testing. The absence of a single diagnostic criterion is largely propagated by the lack of a histopathologic gold standard for PA diagnosis. 

Given the high prevalence of PA, and particularly, the more recent recognition that milder forms of PA may be common even when there are is no radiographic evidence of adrenal neoplasia, a key issue is to understand what may underlie the pathogenesis of PA. The use of specific CYP11B2 antibodies has revealed the presence of aldosterone producing cell clusters (APCCs) in a remarkable proportion of morphologically normal adrenal glands [32,33]. APCCs have been described as non-neoplastic foci of CYP11B2 staining that can co-exist adjacent to aldosterone-producing adenomas, extend into zona fascisculata, and often harbor known somatic mutations in aldosterone driver genes [32,34,35]. Collectively, this evidence strongly suggests that APCCs may represent a common aldosterone-secretory abnormality, which can be detected in normotensives with normal adrenal glands [36], may increase in number with older age, and may thus be a pre-neoplastic and age-dependent precursor to more overt PA [37]. 

Along with the finding of APCCs, our understanding of the genetics of PA has undergone substantial revision in the past decade. These advances have been described in detail in recent reviews, and therefore, we will summarize them briefly herein. The first described inheritable form of PA is glucocorticoid remediable aldosteronism (GRA), also known as familial hyperaldosteronism type I (FH-I). The estimated prevalence of GRA is only 1% of PA subjects and is secondary to a chimeric gene with recombination of 11β-hydroxylase (*CYP11B1*) and aldosterone synthase (*CYP11B2*) genes [17,38]. The chimeric fusion results in regulation of aldosterone synthesis by adrenocorticotropic hormone (ACTH) and can be mitigated by suppressing ACTH with glucocorticoids. GRA should be considered in patients with early-onset hypertension and family history of PA or early-onset cerebrovascular accidents [17]. GRA has considerable variation in its clinical presentation, some families do not manifest with the classic hypertension and hypokalemia, and therefore, the gold standard diagnosis is sequencing to confirm the chimeric *CYP11B2*/*CYP11B1* gene [39].

Familial Hyperaldosteronism type II (FH-II) has been described in 6% of PA cases but is by definition a familial form of PA with yet unknown genetic loci [17]. Whether the discovery of newer inheritable forms of PA (below) results in a reclassification of FH-II remains to be seen. 

Most recently, a new Familial Hyperaldosteronism named type III (FHIII), was described secondary to a gain-of-function germline mutation in the *KCNJ5* gene [40,41]. The *KCNJ5* gene mutation results in loss of potassium selectivity in a zona glomerulosa potassium channel, consequent increased influx of sodium resulting in a higher cell membrane potential and lower depolarization threshold, and therefore increased aldosterone synthesis and secretion [17,38,41]. Interestingly, although germline mutations in *KCNJ5* are rare, somatic mutations of *KCNJ5* have been described in nearly half of aldosterone-producing adenomas [38]. In addition, somatic (and rarely germline) mutations in other zona glomerulosa channels that result in increased cell membrane potential and decreased depolarization thresholds have been described in *CACNA1D* gene (codes for calcium channel, voltage-dependent, L-type, α-1d subunit), *CACNA1H* gene (codes for T-type voltage dependent calcium channel Cav3.2) [42], while somatic mutations have been found in the *ATP1A1* gene (Na^+^, K^+^-ATPase), and *ATP2B3* gene (calcium transporting ATPase 3) [38,43].

Treatment of PA should be tailored according to the severity of disease, age of the patient, anatomic type of disease (unilateral adenoma versus bilateral hyperplasia) and desire for surgery. Laparoscopic surgery is the recommended and ideal therapeutic intervention if PA is unilateral since it can cure aldosterone excess and improve long-term cardiometabolic outcomes and blood pressure control [16,19,44]. In most cases of PA, the source of autonomous aldosterone is bilateral or surgery is not pursued due to other complicating factors, and therefore, medical therapy with MR antagonists (such as spironolactone and eplerenone) is recommended [16]. Although medical therapy is often assumed to be equally efficacious to surgical therapy if blood pressure is normalized, a recent study suggested that this assumption may not be correct. In this large cohort study, patients with PA treated with MR antagonists had 2–3 times higher risk for incident cardiovascular events and death, when compared to age-matched essential hypertensives, even though they had similarly controlled (and relatively normalized) blood pressure while on MR antagonist therapy [19]. In contrast, PA patients who developed a longitudinal increase in renin activity while being treated with MR antagonists had the same risk for incident cardiovascular events and death as patients with essential hypertension, suggesting that the excess risk in medically treated PA may be due to inadequate MR blockade as manifested by a persistently suppressed renin. Future prospective studies will be needed to determine the optimal approach for medical therapy in PA, and how it compares with surgical adrenalectomy [19].

## 3. Hypercortisolism

Endogenous hypercortisolism, with or without the overt manifestations of Cushing syndrome, can result in chronic stimulation of the glucocorticoid receptor and also potentially the MR, with consequent development of hypertension, insulin resistance, diabetes, and cardiovascular disease and mortality [45,46,47,48,49].

There are several potential mechanisms for developing the LRH phenotype with hypercortisolism. Activation of the glucocorticoid receptor by excess cortisol can induce a direct vasopressor effect and elevations in blood pressure [50]. However, cortisol-mediated activation of the renal MR can also play a role in developing LRH. Cortisol and aldosterone are similarly potent MR agonists, but cortisol is inactivated to cortisone by 11β-hydroxysteroid dehydrogenase Type 2 (11βHSD2), thereby “protecting” the renal MR from abundant cortisol stimulation and permitting a high-affinity aldosterone-MR interaction. However, in states of severe hypercortisolism, excess cortisol can overwhelm 11βHSD2 activity and result in direct cortisol-mediated MR activation and subsequent intravascular volume expansion with suppression of renin and aldosterone [50]. Thus, the LRH phenotype with hypercortisolism is unique from PA in that it is a hyporeninemic hypoaldosteronism manifesting with Cushing syndrome and apparent MR overactivation (i.e., hypertension, hypokalemia and increased kaliuresis) [47,51,52]. Studies have also shown that chronic hypercortisolism can also result in activation of ENaC and increases in angiotensinogen [50,51,53].

Endogenous hypercortisolism is most commonly due to a benign pituitary ACTH secreting tumor (Cushing disease), and less frequently due to benign or malignant adrenal tumors (ACTH-independent hypercortisolism), and non-pituitary ACTH-secreting tumor (ectopic ACTH secretion). Current guidelines recommend that the diagnosis of overt hypercortisolism be confirmed using two distinct tests: elevated late night salivary cortisols, elevated 24 h urinary free cortisols, and/or incomplete suppression of cortisol following overnight dexamethasone [47]. Beyond overt hypercortisolism, the concept of “subclinical hypercortisolism” or “autonomous cortisol secretion” in association with adrenocortical adenomas, whereby there is excess cortisol without the hallmark signs of Cushing syndrome, is being recognized as a prevalent phenotype that is associated with higher risk for cardiometabolic disease [54]. For example, studies using mass spectrometry to analyze steroid metabolites have shown that even apparently nonfunctional adrenal adenomas secrete higher concentrations of glucocorticoids when compared to patients with no adenomas [55]; therefore, it is possible that many or most adrenocortical neoplasms secrete at least miniscule amounts of glucocorticoid, and that the categorization of “nonfunctional adenoma” may be misleading or a misnomer. In parallel, cohort studies in patients with incidentally discovered benign adrenocortical tumors have observed that patients with subclinical hypercortisolism have a higher risk for incident cardiovascular disease and death when compared to those with “nonfunctional” tumors [49]. Further, patients with apparently “nonfunctional” adrenocortical tumors have a higher risk for incident diabetes, a risk that was related to the degree of autonomous cortisol secretion within the normal range (normal defined as cortisol < 1.8 mcg/dL following overnight 1 mg dexamethasone suppression), compared with patients with no adrenal tumors [48]. Thus, as with PA, there is increased recognition that adrenal hypercortisolism is not a categorical phenotype, rather exists over an expanded continuum ranging from mild and subclinical to overt Cushing syndrome, with a parallel risk profile of increasing cardiometabolic risk. Future and ongoing studies will be necessary to determine when intervention to treat the autonomous cortisol is indicated with respect to abrogating incident adverse outcomes.

The genetics of hypercortisolism, both related to pituitary and adrenals tumors, has undergone dramatic change in the recent decade. Concerning pituitary tumors, germline mutations that cause Cushing disease have been previously ascribed to *MEN1* gene mutations and *AIP* mutations in Familial isolated pituitary adenomas (FIPA). More recently, next generation sequencing of pituitary adenomas identified somatic driver mutations in ubiquitin-specific protease 8 (*USP8*), leading to ACTH excess and hypercortisolism [56]. In adrenal hypercortisolism, novel genes have also been described beyond the classic germline mutations of the Carney complex (*PRKAR1A* gene, regulatory subunit of protein kinase) or McCune-Albright syndrome (activating somatic mutations in *GNAS1* oncogene) [56]. Germline mutations in *ARMC5* (Armadillo repeat-containing protein 5) have been identified in familial cases and are present in approximately 50% of sporadic cases of macronodular adrenal hyperplasia [57]. In addition, somatic events in *ARMC5* and *PRKACA* (encodes for catalytic subunit α of protein kinase A) are frequently observed in cortisol-producing adrenal adenomas [38,58,59]. 

## 4. Apparent Mineralocorticoid Excess Syndrome

The syndrome of apparent mineralocorticoid excess (AME) is a rare disease, first described in the late 1970s, as a syndrome of severe pediatric LRH [60]. The AME syndrome is an autosomal recessive condition due to loss of function mutations in 11βHSD2. The insufficient activity of 11βHSD2 permits normal cortisol concentrations to activate the renal MR, resulting in a syndrome of MR-mediated LRH, low aldosterone, hypokalemia, alkalosis, and usually failure to thrive and poor weight gain.

The diagnosis of AME is usually suspected in the setting on non-aldosterone dependent LRH with classic features of MR activation and confirmed by a high cortisol/cortisone (F/E) ratio in the serum or urine, and/or genetic sequencing of 11βHSD2 [61,62]. These infrequent cases of classic AME are treated with low dose dexamethasone to suppress endogenous ACTH and cortisol (since dexamethasone is not metabolized by 11βHSD2) in combination with an MR antagonist, and in extreme cases, renal transplantation [63].

Although the classic AME syndrome is rare, recent research suggests that the spectrum of cortisol-mediated MR activation may be more expansive than currently recognized in that milder forms of AME may be common. For instance, Ulick et al. described a milder version of AME and named it the type 2 variant, caused by a decrease in the cortisol clearance rate but not related to cortisone conversion [64]. More recently, cross-sectional human studies have shown that both cortisone levels and renin activity decline with older age, suggesting a potential age-dependent decline in the activity of 11βHSD2 [65]. Since higher F/E ratio and the low-renin phenotype have been correlated with higher blood pressure in both adults and children [66,67], new evidence suggests that a proportion of LRH may be explained by a less severe, or “non-classical”, phenotype of AME that may respond to MR antagonists [68]. Interestingly, milder phenotypes of AME may be explained by less severe inactivating mutations or heterozygosity with partial activity of 11βHSD2, but also by consumption of exogenous inhibitors of 11βHSD2such as licorice or grapefruit [69]. In a recent study, we observed that lower cortisone levels (in combination with higher F/E ratio) were strongly associated with higher MR activity (lower renin activity and higher urinary potassium excretion) in patients suspected to have mild or non-classical AME (Tapia-Castillo, Baudrand, Vaidya, et al. personal communication). The summary of available data suggest that beyond the rare classical phenotype of AME, milder forms of non-classical AME may contribute to LRH and may be best detected by recognition of high F/E ratio in combination with low cortisone levels. 

## 5. Congenital Adrenal Hyperplasia

Congenital adrenal hyperplasia (CAH) is one of the most common autosomal recessive disorders caused by mutations in genes involved in cortisol biosynthesis enzymes. More than 90% of described cases of CAH are 21-hydroxylase deficiency. Less common, and pertinent to the LRH phenotype, are CAH syndromes due to 11β-hydroxylase [P450c11β] and 17α-hydroxylase [P450c17] deficiency.

CAH caused by steroid 11β-hydroxylase deficiency is considered a rare recessive disorder, with an overall frequency of 1/100,000 live births. Unlike 21-hydroxylase CAH that is more frequent in Europe and North America, 11β-hydroxylase deficiency is more frequently described in the Middle East and Africa [70]. The gene *CYP11B1* encodes 11β-hydroxylase that catalyzes the conversion of 11-deoxycortisol and 11-deoxycorticosterone to cortisol and corticosterone in the zona fasciculata. Thus, from a clinical perspective, deficiency of 11β-hydroxylase, results in low levels of cortisol and high levels of 11-deoxycortisol and 11-deoxycorticosterone (DOC) and a shunting of metabolites into the androgen synthesis pathway. Depending on the severity of the mutations and 11β-hydroxylase deficiency, patients may present with signs of hyperandrogenism (masculinization of genitalia, hirsutism, premature bone maturation and precocious puberty) and excessive MR activation due to increased DOC (hypertension and hypokalemia) [70]. The increased activation of the MR leads to sodium reabsorption, increased intravascular volume expansion, and consequently LRH. The diagnosis can be confirmed via elevated DOC and androgen levels (with or without cosyntropin stimulation) and/or genetic sequencing of 11β-hydroxylase. Treatment involves the use of MR antagonists in addition to glucocorticoids in order to suppress ACTH.

17α-hydroxylase deficiency is another rare form of CAH and results as a consequence of inactivating mutations in the *CYP17A1* gene that regulates steroid 17-hydroxylation followed by the 17,20-lyase reactions. The most common presentation of 17α-hydroxylase deficiency is an adolescent female with absence of secondary sexual characteristics, amenorrhea, and LRH [71]. The 17α-hydroxylase deficiency phenotype is explained by impaired steroidogenesis in both the adrenals and the gonads: abnormal cortisol synthesis, elevation of DOC and resultant MR-mediated LRH, and impaired sex-steroid synthesis. These patients typically display low cortisol, but high levels of corticosterone, so adrenal crises are rarelyobserved. LRH is due to high levels of DOC that result in MR activation, hypertension, and hypokalemia [72], and treatment involves MR antagonists to normalize blood pressure and replacement of sex steroids [72]. 

## 6. Liddle Syndrome

The Liddle syndrome is a rare autosomal dominant monogenic disease caused by gain-of-function mutations of subunits of the epithelial sodium channel (ENaC). Since increased luminal ENaC in the distal nephron is the common final mechanism for MR-mediated sodium reabsorption, Liddle syndrome resembles states of mineralocorticoid excess. The classical clinical presentation is severe hypertension in a young patient, with hypokalemia and metabolic alkalosis, in the setting of suppressed renin; however, unlike PA, aldosterone levels are low or undetectable and the syndrome does not improve with MR antagonist therapy [73,74,75,76]. The diagnosis of Liddle syndrome can be confirmed by genetic sequencing of the *SCNN1B* and *SCNN1G* genes, which encode for the β and γ subunits of ENaC [75], or the *SCNN1A* gene [77], and the treatment of choice are ENaC inhibitors such as amiloride or triamterene [74].

In a similar theme to the aforementioned discussions on PA, hypercortisolism, and AME, Liddle syndrome may exist across a more extensive spectrum than currently recognized. “Classical” Liddle syndrome is rare and manifests with a severe phenotype; however, recent studies have suggested that a substantial proportion of LRH patients also have a low serum aldosterone [13,24]. Although there are potentially many mechanistic explanations for a phenotype of hypertension with low renin and aldosterone, a recent clinical trial demonstrated that treating African patients with a low-renin and aldosterone phenotype using ENaC inhibitors resulted in the most effective control of blood pressure [78], a finding that extended genetic studies demonstrating a high prevalence of *SCNN1B* variants in individuals of African descent with LRH [79].

## 7. Glucocorticoid Resistance Syndrome

Inactivating mutations of the glucocorticoid receptor gene (*NR3C1*) in chromosome 5q31–q32 cause familial glucocorticoid resistance [80,81,82]. All severe described cases are secondary to mutations that affect the function of either the ligand binding or the DNA binding domain. This is a rare syndrome, inherited either as an autosomal recessive or dominant disease, that is characterized by the classical biochemical pattern seen in endocrine receptor resistance syndromes: hypercortisolism and increased ACTH but without the classic clinical features of Cushing’s syndrome [81]. Although the increased ACTH and cortisol are necessary to try and overcome glucocorticoid receptor resistance, the undesired effects are ACTH-induced hypersecretion of adrenal mineralocorticoids and androgens, and hypercortisolism-mediated renal-MR activation. Thus, the classic phenotype of glucocorticoid resistance is LRH, hypokalemia, hirsutism in females, alopecia in males, acne, precocious puberty and menstrual irregularities, in addition to chronic fatigue and malaise as a consequence of relative glucocorticoid deficiency [83]. The treatment is usually overnight low dose dexamethasone to suppress ACTH, and thus improving ACTH-induced hypercortisolism, excess mineralocorticoids, and hyperandrogenism. The addition of spironolactone can further help to control hypertension and hirsutism in females as well. Interestingly, haploinsufficiency of the GR receptor can also present with LRH due to MR activation by elevated cortisol rather than increased DOC as observed in classical glucocorticoid resistance [84].

## 8. Gordon Syndrome

Gordon Syndrome (GS) was described in the 1960s and is a very rare familial hypertension syndrome that presents with low renin and hyperkalemia. GS is considered to have autosomal dominant inheritance, although new molecular studies suggest some recessive cases [85]. Mutations in *WNK1*, *WNK4*, *CUL3* and *KLHL3* genes have all been identified in GS [85], due their interaction with the thiazide sensitive Na/Cl cotransporter (NCC) in the distal nephron, responsible for sodium reabsorption, but also reducing cell-surface expression of renal outer medullary potassium channel (ROMK), explaining the lower potassium excretion in the collecting ducts [86]. 

Subjects with GS have suppressed renin levels consistent with their high volume state, with relatively low aldosterone levels (that may still fall in the reference range) despite their hyperkalemia, that can be occasionally severe and with periodic paralysis [86]. Both hypertension and hyperkalemia can be reversed by dietary sodium restriction and/or low doses of thiazide diuretics.

## 9. MR Activating Mutations

This is a novel entity secondary to a gain of function mutation, by a substitution of leucine for serine at codon 810 (abbreviated as S810L), in the MR gene. The S810L mutation was recently described and is responsible for an MR-mediated LRH that has also been described as worsening during pregnancy [87]. The worsening in pregnancy is explained by the activation of the mutant S810L MR by progesterone, where progesterone typically antagonizes wild-type MR. In males and non-pregnant females, cortisone and 11-dehydrocorticosterone (cortisol and corticosterone metabolites respectively) can activate the mutant MR and result in increased sodium reabsorption [87]. Spironolactone is surprisingly ineffective since it has agonist properties on the mutant MR and can paradoxically increase MR activation [88]. Recently, Amazit et al. described that in addition to amiloride to inhibit ENaC, the novel and potent nonsteroidal selective MR antagonist finerenone has MR antagonistic properties and may therefore represent a useful treatment option [89]. 

## 10. Proposed Approach to the Patient with Low-Renin Hypertension

Prior to considering an elaborate differential diagnosis for LRH, it is important to confirm the phenotype. First, factors than can reversibly suppress renin include, increased intra-vascular volume, certain medications (see below), supine posture, menstrual phase and high dietary sodium intake, and therefore must all be considered [16,90,91]. The diagnosis of LRH inherently assumes a chronic phenotype, and therefore, it is important to ensure that a single measure of low renin was not induced by a confounding or transient factor. Second, it is important that renin levels are measured in a reliable laboratory using a validated technique. Renin activity assays are increasingly being measured via LC-MS/MS and the global forecast suggests that commercial renin activity assays may be largely replaced by assays of renin concentration [92]. To avoid misinterpretation, the most ideal confirmation of LRH entails measurement of renin in a seated posture, while on unrestricted dietary sodium intake, during the follicular phase of the menstrual cycle (in women), and ideally without the confounding effects of MR antagonists, β-blockers, diuretics, angiotensin-converting enzyme inhibitors, or angiotensin receptor blockers [16,18,93]. Although most patients in industrialized countries consume sufficient sodium to maintain a dietary sodium balance of greater than 100–150 mmol/24 h, it has been shown that severe dietary sodium restriction (<40 mmol/24 h) can raise renin even among patients with overt PA; thus, emphasizing the importance of recommending unrestricted dietary sodium intake when phenotyping renin status, and consideration of confirming 24 h urinary sodium balance [94].

**Figure 1:**
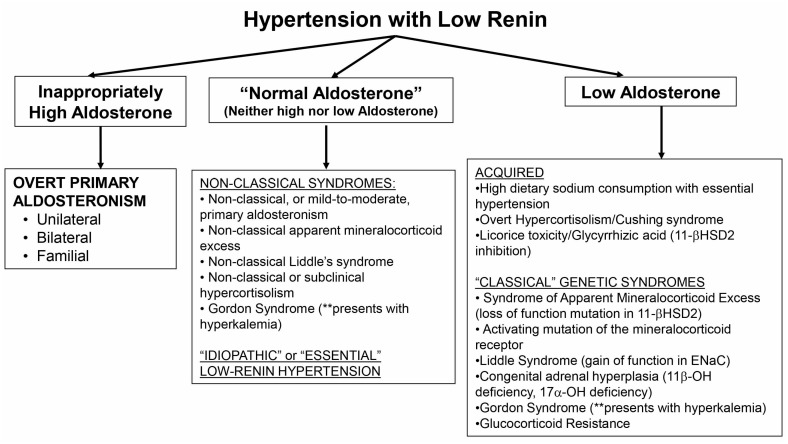
A proposed approach to the patient with low-renin hypertension. ENaC = epithelial sodium channel; 11-βHSD2 = 11-β hydroxysteroid dehydrogenase type 2; 11β-OH = 11-β hydroxylase; 17α-OH = 17-α hydroxylase.

The exact definition of what constitutes low renin is not well established. Suppression of renin below the limit of detection is probably the most commonly used definition, however, in non-clinical research settings, more elaborate physiological maneuvers have been employed to characterize a phenotype of low renin or a renin that does not adequately stimulate to physiological provocation [4,27,94,95,96]. Once there is confidence that the phenotype represents LRH, the examination of aldosterone levels in the context of renin suppression can provide insight into potential underlying mechanisms of disease (Figure 1). An inappropriately high aldosterone level narrows the differential diagnosis to PA. The most severely elevated aldosterone levels (>15–20 ng/dL) require little confirmation, and similarly, even levels > 10 ng/dL are highly suggestive among patients with LRH that can be confirmed using a variety of confirmatory tests (sodium or fludrocortisone suppression or captopril challenge). However, milder forms of PA are common and may not exhibit marked elevations in aldosterone or blood pressure [22,23,24,25], and therefore, these non-classical instances of PA may represent missed opportunities at mitigating the age-dependent cardiometabolic consequences of autonomous aldosterone secretion [10,11]. Future studies, and a renewed commitment to better understanding the true prevalence and severity spectrum of PA, will be needed to identify new and efficient methods for identifying non-classical PA, including novel biomarkers of MR activation [97].

A phenotype of LRH whereby aldosterone levels are clearly suppressed or very low generates a broad consideration of underlying mechanisms. Acquired or non-genetic etiologies include essential (or idiopathic) hypertension in the context of high dietary sodium intake, as well as severe hypercortisolism or reversible inhibition of 11BHSD2 (Figure 1). The classical genetic syndromes that result in a low renin and aldosterone phenotype are rare, and include classical AME, activating mutations in the MR or ENaC (Liddle syndrome), atypical forms of CAH, Gordon syndrome, and glucocorticoid resistance syndrome. The default explanation for LRH with low aldosterone, once these infrequent and classical diseases have been excluded, is essential hypertension with high dietary sodium consumption.

Finally, a great deal of progress has been made in identifying “non-classical” phenotypes that represent a milder continuum of classical disease states. These non-classical conditions should be considered when aldosterone levels are normal or not in the extremes: neither markedly elevated nor remarkably suppressed. Mild and moderate variants of PA, hypercortisolism, AME, and potentially even Liddle’s syndrome, may be common and the early recognition and treatment of these phenotypes may prevent years of untreated hypertension and cardiovascular risk. Gordon’s syndrome may also present with this phenotype. MR antagonists have already been shown to be the most effective add-on therapy in low-renin resistant hypertension [98], however, the adoption of MR antagonists as effective anti-hypertensives that can be used early in the treatment of hypertension using a renin- and aldosterone-based phenotyping approach has not been evaluated with sufficient evidence for widespread recommendation. Non-classical PA, hypercortisolism, and non-classical AME may all potentially respond well to MR antagonists, and therefore, future studies are needed to evaluate the efficacy of early initiation of MR antagonists in the treatment of LRH to formulate evidence-based clinical practice recommendations.
